# Regulation of hyperoxia-induced social behaviour in *Pristionchus pacificus* nematodes requires a novel cilia-mediated environmental input

**DOI:** 10.1038/s41598-017-18019-0

**Published:** 2017-12-14

**Authors:** Eduardo Moreno, Bogdan Sieriebriennikov, Hanh Witte, Christian Rödelsperger, James W. Lightfoot, Ralf J. Sommer

**Affiliations:** Department for Integrative Evolutionary Biology, Max Planck Institute for Developmental Biology, 72076 Tübingen, Germany

## Abstract

Social behaviours are frequently utilised for defence and stress avoidance in nature. Both *Caenorhabditis elegans* and *Pristionchus pacificus* nematodes display social behaviours including clumping and bordering, to avoid hyperoxic stress conditions. Additionally, both species show natural variation in social behaviours with “social” and “solitary” strains. While the single solitary *C. elegans* N2 strain has evolved under laboratory domestication due to a gain-of-function mutation in the neuropeptide receptor gene *npr-1*, *P. pacificus* solitary strains are commonplace and likely ancestral. *P. pacificus* therefore provides an opportunity to further our understanding of the mechanisms regulating these complex behaviours and how they evolved within an ecologically relevant system. Using CRISPR/Cas9 engineering, we show that *Ppa-npr-1* has minimal influence on social behaviours, indicating independent evolutionary pathways compared to *C. elegans*. Furthermore, solitary *P. pacificus* strains show an unexpected locomotive response to hyperoxic conditions, suggesting a novel regulatory mechanism counteracting social behaviours. By utilising both forward and reverse genetic approaches we identified 10 genes of the intraflagellar transport machinery in ciliated neurons that are essential for this inhibition. Therefore, a novel cilia-mediated environmental input adds an additional level of complexity to the regulation of hyperoxia-induced social behaviours in *P. pacificus*, a mechanism unknown in *C. elegans*.

## Introduction

Nematodes have a highly developed chemosensory system and are able to respond to a multitude of environmental cues. These cues can be associated with food, stress conditions and also other animals with whom they can have phoretic, necromenic or parasitic interactions^1,2^. Some of the behavioural responses to chemosensory cues are increasingly recognized to be associated with substantial natural variation, such as the intensely studied so-called “social feeding behaviours” clumping and bordering in *Caenorhabditis elegans*. Clumping is the aggregation of nematodes in feeding groups under laboratory conditions, which occurs in all wild isolates of *C. elegans*. Clumping correlates with bordering behaviour and indicates the preference of the worms for the border of the bacterial lawn over the centre. Both behaviours are found together in so-called “social strains”. In contrast, strains that do not exhibit clumping and bordering behaviours and instead display a solitary feeding behaviour have been called “solitary strains”; for example, the *C. elegans* laboratory reference strain N2^3,4^.

The terms “social” and “solitary” provide a simple way to differentiate between clumping//bordering *vs*. non-clumping/bordering strains. However, these terms oversimplify the nature of these complex behaviours. It has been suggested that clumping and bordering behaviours are instigated to avoid the hyperoxic stress conditions induced by the 21% oxygen concentration [O_2_] present in the laboratory^4^, since both behaviours result in the nematodes being exposed to lower [O_2_]. Clumping lowers the [O_2_] due to increased consumption by the more tightly clustered nematodes^5^, while via bordering the nematodes are exposed to lower [O_2_] created by the boundary of the bacterial lawn being thicker than that of the centre and thus consuming more oxygen^4^. Therefore, “hyperoxia-avoidance behaviours” is a more accurate term for these behaviours. However, the original terms “social” and “solitary” will be utilised in this work for consistency with previous literature.

The solitary foraging behaviour of the N2 strain has evolved in the laboratory from ancestors adapted towards 21% [O_2_] avoidance in their natural habitats (soil, compost and rotten fruits)^6,7^. Specifically, this solitary foraging behaviour is the result of a *gain-of-function* mutation in the neuropeptide Y-like receptor encoded by the *npr-1* gene, which creates a hyperactive neural circuit with pleiotropic effects on a wide variety of other life-history traits^3,8–12^. The two alleles of *npr-1* in *C. elegans* differ at codon 215, with social strains encoding for the ancestral *npr-1F* (phenylalanine) allele and N2 encoding *npr-1V* (valine)^3^. The standard lab husbandry of *C. elegans* has strongly selected for the *npr-1 215* *V* allele and the associated solitary feeding behaviour, which has been retained through clonal propagation during N2 maintenance^7^.

**Figure 1:**
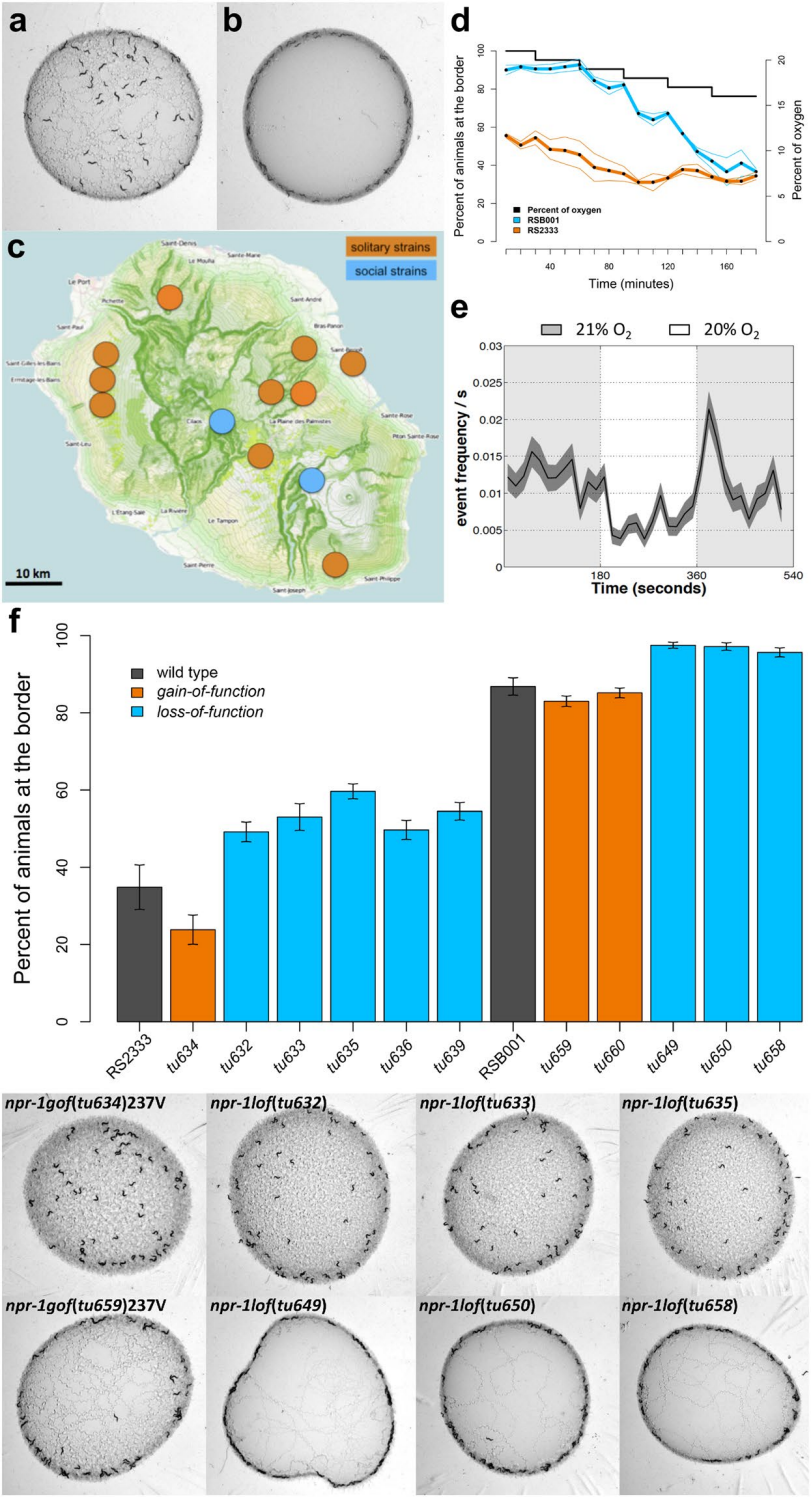
Natural variation of social behaviours in *P. pacificus* independent of *Ppa-npr-1*. **(a)** Clumping/bordering assay for the *P. pacificus* reference strain RS2333, which shows a solitary behaviour under laboratory conditions, characterized by low levels of both clumping and bordering behaviours. (**b)** Clumping/bordering assay for the *P. pacificus* RSB001 strain, isolated at 2327 m.a.s.l. on La Réunion Island, which shows strong clumping and bordering behaviours under laboratory conditions. (**c)** Map of La Réunion Island showing the locations from which *P. pacificus* nematode strains have been isolated. Social strains, belonging to the phylogenetic lineage B, have been isolated from only two locations above 2000 m.a.s.l. (modified from OpenStreetMap^®^ under the Open Database License (ODbL), © OpenStreetMap contributors (https://www.openstreetmap.org/copyright)). (**d)** Regulation of bordering behaviour by oxygen levels: comparison of the dynamic changes in bordering behaviour between RSB001 and RS2333 strains during oxygen shifts from 21% to 16%, with a 1% decrement occurring every 30 min (modified from Moreno *et al*.^15^). Black dots represent the mean and thin lines represent the standard error of the mean (SEM). (**e**) Ω-turn rate response of RS2333 to 21% −>20% −>21% oxygen shifts on a lawn of *Escherichia coli* OP50^15^. The black line represents the mean Ω-turn and the grey area, the SEM. (**f)** Bordering behaviour of RS2333, RSB001 and the *Ppa-npr-1* mutants generated using CRISPR/Cas9 system (Suppl. Table S1). In all bar-plots arrows represent the standard error of the mean (SEM). For statistical analysis see Suppl. Table S2.

The nematode *Pristionchus pacificus* has been established as a model for evolutionary studies in comparison to *C. elegans* ^13,14^. In addition, the ecology and population genetics of *P. pacificus* have by now been well characterised and have contributed to its establishment as a model for integrative eco-evo-devo studies^14^. Further to this, many of its behaviours are now being dissected including its foraging behaviour as *P. pacificus* also shows natural variation in foraging behaviours similar to that observed in *C. elegans*. However, it is apparent that the origin of this natural variation is associated with differing evolutionary histories between the two species^15^. In contrast to *C. elegans*, solitary feeding behaviour is widespread among *P. pacificus* natural isolates (Fig. 1a) and the molecular phylogeny of *P. pacificus* strains suggests this pattern to be ancestral^15^. However, all strains of the phylogenetic clade B of *P. pacificus* display social behaviours under laboratory conditions^15^ (Fig. 1b). This clade is endemic to high-altitude locations (2100–2400 metres above mean sea level, hereafter m.a.s.l.) on La Réunion Island^16^ (Fig. 1c), characterized by an atmospheric oxygen partial pressure of around 16.4 kPa^17^. Since the social behaviours of clade B nematodes at 21% [O_2_] are suppressed when [O_2_] drops to 16% this may reflect a possible adaptation of this clade to high-altitude-associated hypobaric hypoxia found in the wild^15^ (Fig. 1d). Therefore, clade B strains may perform social behaviours to avoid hyperoxic conditions in the laboratory, but they behave also as solitary strains when the O_2_ levels are similar to those that they experience in the wild, suggesting that all *P. pacificus* strains behave solitary in natural conditions. These observations may be related to the necromenic association of *P. pacificus* and its connection to scarab beetle hosts, in which the arrested dauer stage of the nematode colonizes the insect and resumes development only after the death of the beetle by feeding on the proliferating microbes on the beetles’ carcass^18^. The need for the nematodes to search for a new host may have favoured the emergence of a mechanism that induces solitary foraging and ultimately favours dispersion.

Previous QTL analyses indicated that the genetic architecture of social behaviours in *P. pacificus* is more complex than in *C. elegans*, with at least three major QTLs involved, none of which are associated with the *P. pacificus npr-1* locus^15^. Additionally, both the solitary RS2333 and the social RSB001 strains encode the *npr-1F* allele at codon 237, corresponding to the *npr-1F* allele at codon 215 present in social *C. elegans* strains^15^. These observations suggested that *npr-1* is not a major contributor to social behaviours in *P. pacificus*, while a smaller contribution of this gene cannot be ruled out. Here, we disentangled the contribution of *npr-1* to the regulation of the social behaviours in *P. pacificus* by producing both *gain-of-function* (*gof*) and *loss-of-function* (*lof*) mutations using the CRISPR/Cas9 system recently established for *P. pacificus* ^19^.

In addition to clumping and bordering, nematode locomotive behaviours are likewise influenced by environmental oxygen. Hyperoxic stress conditions increase the rate of reorientation movements (omega (Ω) turns) while crawling. The variation in the rate of Ω-turns in response to small shifts from 20% to 21% in [O_2_] correlates with the social behaviours in *C. elegans*. For instance, the social *C. elegans* strain CB4856 increases the rate of Ω-turns when [O_2_] shifts from 20% to 21%, whereas the solitary N2 strain does not modify its Ω-turn rate in response to these shifts^7,20^. This variation reflects a different neuronal sensitivity to [O_2_], dependent on the globin encoding *gbl-5* gene. The *glb-5* N2 variant has also arisen under laboratory conditions and has been spread during N2 maintenance on agar plates^7^.

On the contrary, *P. pacificus* RS2333 shows a surprising and unexpected locomotive response to [O_2_] shifts from 20% to 21%, indicating that nematodes of this strain perceive 21% [O_2_] as hyperoxic stress but do not avoid it by performing social behaviours^15^ (Fig. 1e). Here we show that a similar locomotive response is also observed among solitary strains from La Réunion Island, indicating that this behaviour is not specific to RS2333. It has therefore not been acquired due to any adaptation to laboratory conditions and is a common feature found among solitary *P. pacificus* strains. These findings suggest that solitary strains in *P. pacificus* have acquired a regulatory mechanism that inhibits hyperoxia-avoidance by social feeding. We tested this hypothesis by performing a mutagenesis screen on RS2333, through which we isolated 15 social mutants. By means of whole-genome sequencing and complementation tests we identified four genes encoding intraflagellar transport (IFT) proteins, all of which are involved in the structure and function of neuronal sensory cilia. We subsequently produced mutants in six additional cilia-related IFT genes using the CRIPSR/Cas9 system. The social behaviours displayed by these mutants are regulated by oxygen, similar to the clade B strain and wild type *C. elegans* strains. Our results indicate that *P. pacificus* solitary strains have acquired a novel regulatory mechanism, dependent on environmental sensing through sensory cilia, which prevents social behaviours in response to hyperoxic conditions. Ultimately, these results reveal an additional level of complexity in the regulation of the hyperoxia-induced social behaviours in *P. pacificus* in comparison with *C. elegans*.

## Results

### *Ppa-npr-1* does not play a major role in natural variation of bordering behaviour

Our previous study on the genetic architecture of social behaviours in *P. pacificus* did not provide any evidence for a major contribution of *Ppa-npr-1* to these behaviours although a smaller contribution cannot be ruled out^15^. To investigate the role of *Ppa-npr-1* in the regulation of the social behaviours in *P. pacificus* further, we generated a set of *gof* and *lof* mutants in both, the RS2333 and RSB001 strains, using the CRISPR/Cas9 system (Suppl. Table 1 and Suppl. Fig. S1). sgRNAs were designed to target the first and the sixth exons of the gene to create *lof* mutations (Suppl. Fig. S1a,b,e). In addition, the *gof* mutation that is found in *C. elegans* N2 was induced in both *P. pacificus* strains by co-injection of the sgRNA for exon 6 with a repair template oligonucleotide that recapitulates the change of phenylalanine to valine (Suppl. Table 1 and Suppl. Fig. S1c,d). The social behaviours of these mutants were evaluated by the clumping and bordering assay previously established for *P. pacificus* ^15^. Since both behaviours are correlated in *P. pacificus*, we focused on bordering behaviour as an indicator of the social/solitary character of the strains and mutants along this study.

In the solitary *P. pacificus* RS2333 strain, the *gof* mutation *Ppa-npr-1*(*tu634*)^RS2333^ reduced bordering behaviour even further, although the comparison with the wild type phenotype was not statistically significant (Fig. 1f and Supp. Table S2). More strikingly, a similar *gof* mutation produced in the social RSB001 background *Ppa-npr-1*(*tu659*)^RSB001^ did not induce any observable change in social behaviour (Fig. 1f and Suppl. Table S2). These results indicate that this amino acid substitution either does not induce a solitary phenotype in *P. pacificus*, or has only a minor effect on bordering behaviour.

If *Ppa-*NPR-1 was responsible for the solitary behaviour of *P. pacificus* RS2333, *Ppa-npr-1 lof* mutants produced in this strain should show social behaviour under laboratory conditions. Although we observed a slight increase in the levels of bordering behaviour in five *lof* alleles of RS2333, these levels were still much lower than those typical of social strains (Fig. 1f and Suppl. Table S2). Similarly, an increase in bordering behaviour was observed in three *Ppa-npr-1 lof* alleles generated in the RSB001 background (Fig. 1f and Suppl. Table S2). These results are consistent with *Ppa-npr-1* playing only a minor role in the modulation of bordering behaviour in *P. pacificus*. Importantly, these findings indicate that *Ppa-npr-1* is not responsible for the natural variation observed among wild isolates.

### Hyperoxic stress avoidance is not a result of laboratory domestication in *P. pacificus*

**Figure 2:**
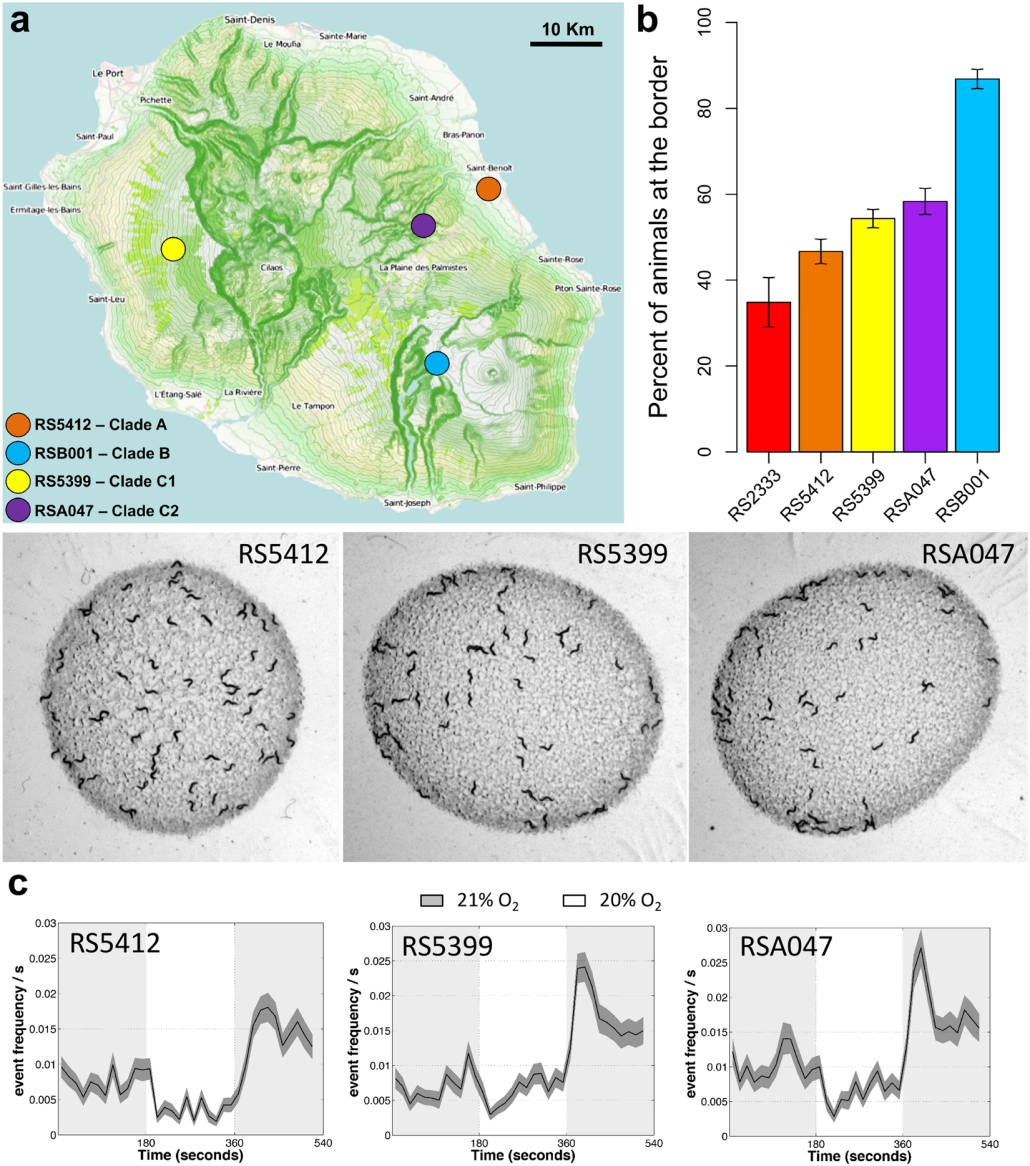
Omega-turn locomotive response in solitary *P. pacificus* strains. **(a)** Locations on La Réunion Island from which the selected solitary strains have been isolated (modified from OpenStreetMap^®^ under the Open Database License (ODbL), © OpenStreetMap contributors (https://www.openstreetmap.org/copyright)). (**b)** Bordering behaviour of selected *P. pacificus* solitary strains. (**c)** Ω-turn rate response to 21% −>20% −>21% oxygen shifts on a lawn of *E. coli* OP50 in selected *P. pacificus* solitary strains. In all graphs, the black line represents the mean Ω-turn and the grey area, the S.E.M. For statistical analysis see Suppl. Table S3.

Our previous study revealed an unexpected locomotive response to [O_2_] shifts from 20% to 21% in RS2333^15^. We therefore, tested whether a similar response is conserved among other solitary strains of *P. pacificus*, focusing on three selected strains isolated from different locations on La Réunion Island, which belong to different phylogenetic clades^15,16^ (Fig. 2a,b). Similarly to RS2333, these strains also modulate their omega-turn rate in response to [O_2_] (Fig. 2c and Suppl. Table S3). These findings indicate that the locomotive response to hyperoxic stress conditions as seen in *P. pacificus* RS2333 has not been a result of domestication in the approximately 3,000 generations this strain is cultured under laboratory conditions, but rather represents a general feature of solitary *P. pacificus* strains under natural conditions.

### Multiple genes control the inhibition of social phenotype in RS2333

**Figure 3:**
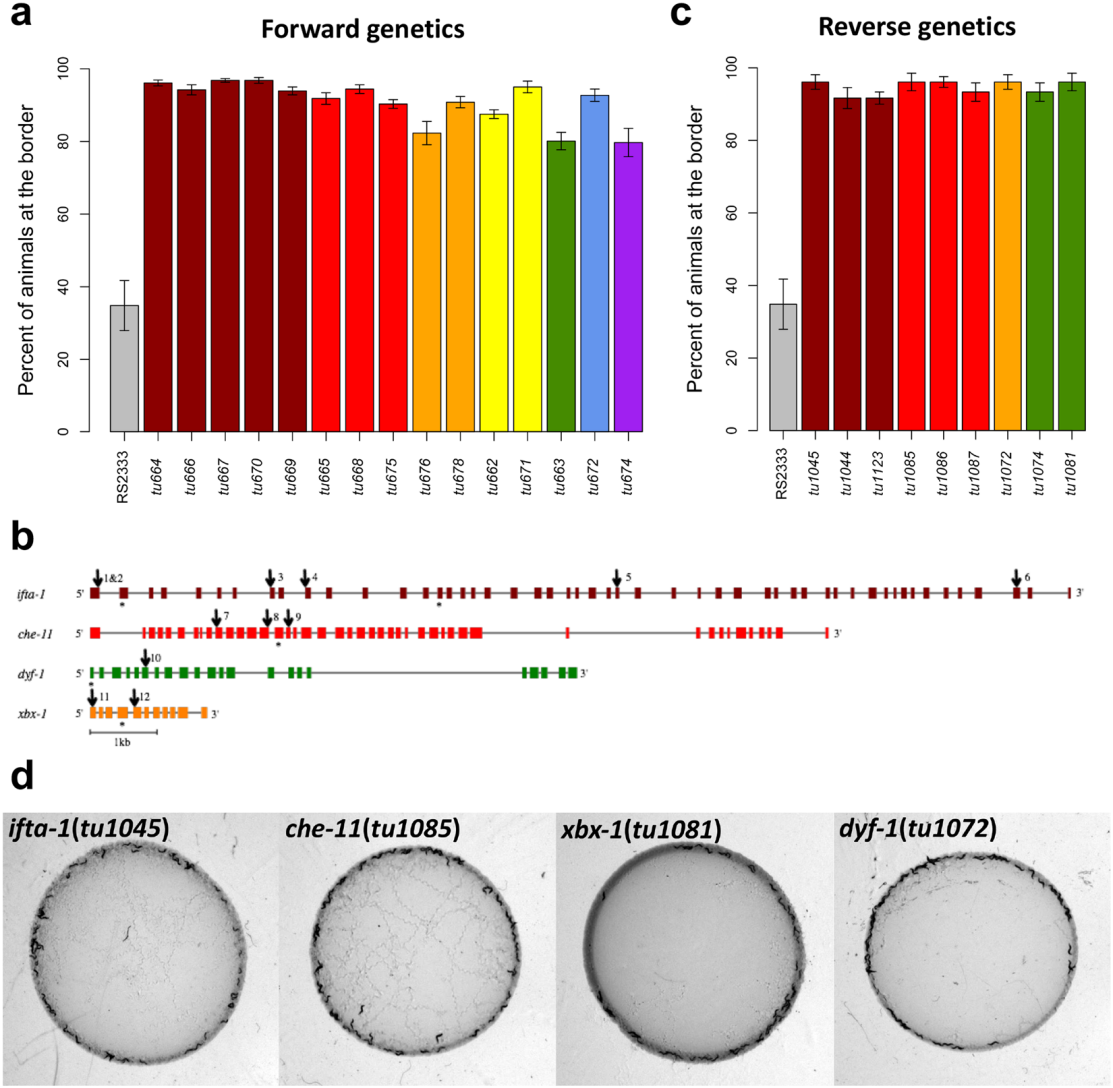
Social behaviours induced by mutations in the *Ppa-ifta-1*, *Ppa-che-11*, *Ppa-dyf-1* and *Ppa-xbx-1* genes. **(a)** Bordering behaviour of RS2333 and the 15 social mutants isolated in forward genetic screens by EMS mutagenesis. Colours indicate complementation groups. For statistical analysis see Suppl. Table S4 and for more information about the mutant alleles see Suppl. Table S5. **(b)** Gene structure of the *Ppa-ifta-1*, *Ppa-che-11*, *Ppa-dyf-1* and *Ppa-xbx-1* genes. Colour boxes indicate exons and solid lines indicate introns. Arrows indicate genetic lesions generated by EMS mutagenesis in mutant alleles (see Suppl. Table S5). Asterisks indicate sgRNA positions. **(c)** Bordering behaviour of RS2333 and mutant alleles of *Ppa-ifta-1*, *Ppa-dyf-1, Ppa-che-11* and *Ppa-xbx-1* produced using CRISPR/Cas9 system (Suppl. Table S6). Colours correspond with genes in Fig. 2b and complementation groups in Fig. 2a. In all bar-plots arrows represent the standard error of the mean (SEM). For statistical analysis see Suppl. Table S7. **(D)** Clumping/bordering assay for representatives of *Ppa-ifta-1*, *Ppa-dyf-1, Ppa-che-11* and *Ppa-xbx-1* mutant alleles.

To explore the potential existence of alternative regulatory mechanisms in *P. pacificus* solitary strains, which block the social behaviours even under hyperoxic conditions, we performed a mutagenesis screen in the RS2333 background searching for social mutants. From a screen of approximately 2,250 gametes we isolated 15 mutants with social behaviours, indicating a mutation frequency of approximately 1 in 150 gametes for this phenotype. Mutant lines were characterised according to the strength of the social phenotype and complementation tests were performed by pairwise crosses, which allowed the identification of seven complementation groups (Fig. 3a and Suppl. Table S4). Thus, social behaviours can be readily induced by mutations in many complementation groups, although its occurrence in nature is restricted to wild isolates from a single phylogenetic lineage on La Réunion Island.

### Mutations in genes encoding intraflagellar transport proteins cause social behaviours

To identify causative mutations, we sequenced the genomes of all mutant lines using an Illumina platform. We used bioinformatic procedures to search for genes sharing SNPs in coding regions according to the complementation groups previously established. This methodology allowed us to identify candidate genes in three complementation groups: i) *tu664*, *tu666*, *tu667*, *tu669* and *tu670* shared mutations in the coding region of the gene contig6-snap.265; ii) *tu665*, *tu668* and *tu675* shared mutations in coding region of contig31-snap.23; and iii) *tu676* and *tu678* share mutations in coding region of contig56-snap.118 (Suppl. Table S5). However, we could not find a common mutation in the mutant alleles of the fourth complementation group (*tu662* and *tu671*), probably due to assembly errors in our reference genome.

Based on combination of automated and manual orthology prediction methods, we found that Contig6-snap.265 corresponds to *Ppa-ifta-1*, which encodes for an evolutionary conserved protein required for retrograde intraflagellar transport (IFT) along ciliary axonemes^21^. IFT refers to the movement of protein complexes called IFT-trains along the axonemes that constitute the cilia of all eukaryotic cells, an essential process for the proper assembly of the cilia and its function in motility, sensing and signalling^22–24^. In *C. elegans* IFTA-1 is associated with the IFT-A sub-complex, which consists of at least six proteins including CHE-11^21^. Indeed, the second gene we identified, contig31-snap.23, is the *P. pacificus* ortholog of *che-11*. In *C. elegans*, CHE-11 is required for the proper incorporation of IFTA-1 into the IFT machinery assembly^21^. Finally, the third identified gene, contig56-snap.118, corresponds to *Ppa-xbx-1*, which encodes for the light intermediate dynein chain equally required for retrograde IFT in *C. elegans* ^25^.

Next, we used a reverse genetic approach by CRISPR/Cas9 engineering to obtain additional alleles in *Ppa-ifta-1*, *Ppa-che-11* and *Ppa-xbx-1* (Fig. 3b, Suppl. Table S6 and Suppl. Fig. S2). In total, we generated seven mutant lines in the RS2333 background, all of which showed very strong social behaviours (Fig. 3c,d and Suppl. Table S7). Finally, we performed complementation tests between the original mutants isolated in the mutagenesis screen and the CRIPSR/Cas9-generated mutants. The F1 progeny derived from these crosses failed to rescue the original solitary phenotype, confirming that the mutations in these genes are responsible for the social behaviour. In summary, we found that the three genes encoding IFT proteins *Ppa-ifta-1*, *Ppa-che-11* and *Ppa-xbx-1*, result in strong social behavioural phenotypes, suggesting a role for sensory cilia in the regulatory mechanisms that prevent social phenotypes in *P. pacificus* RS2333.

### A single allele of *Ppa-dyf-1* shows similar social behavioural phenotypes

Next, we searched among the list of candidate mutations in the three remaining mutant alleles *tu663*, *tu772* and *tu674*, looking for other candidate genes involved in IFT. Indeed, for *tu663* we found a mutation in the gene contig10-snap.384 (Suppl. Table S5), the *P. pacificus* ortholog of *dyf-1*. *dyf-1* encodes an evolutionary conserved protein required for docking the kinesin OSM-3 onto anterograde IFT transport particles^26^. To test whether the mutation in *Ppa-dyf-1* is the causative mutation of the social phenotype of *tu663*, we generated two additional alleles using the CRISPR/Cas9 system in RS2333, *tu1074* and *tu1081* (Fig. 3b, Suppl. Table S6 and Fig. S2), which also showed very strong social behaviours (Fig. 3c,d and Suppl. Table S7). In addition, complementation tests between these alleles and *tu663* confirmed that the mutation in *Ppa-dyf-1* is responsible for the social phenotype of *tu663*. Taken together, our forward genetic screen for mutants with social phenotypes in the solitary RS2333 background has resulted in the isolation of 15 mutants in seven complementation groups, four of which encode for IFT proteins.

### Mutations in genes encoding other IFT proteins also cause social phenotypes in *P. pacificus*

**Figure 4:**
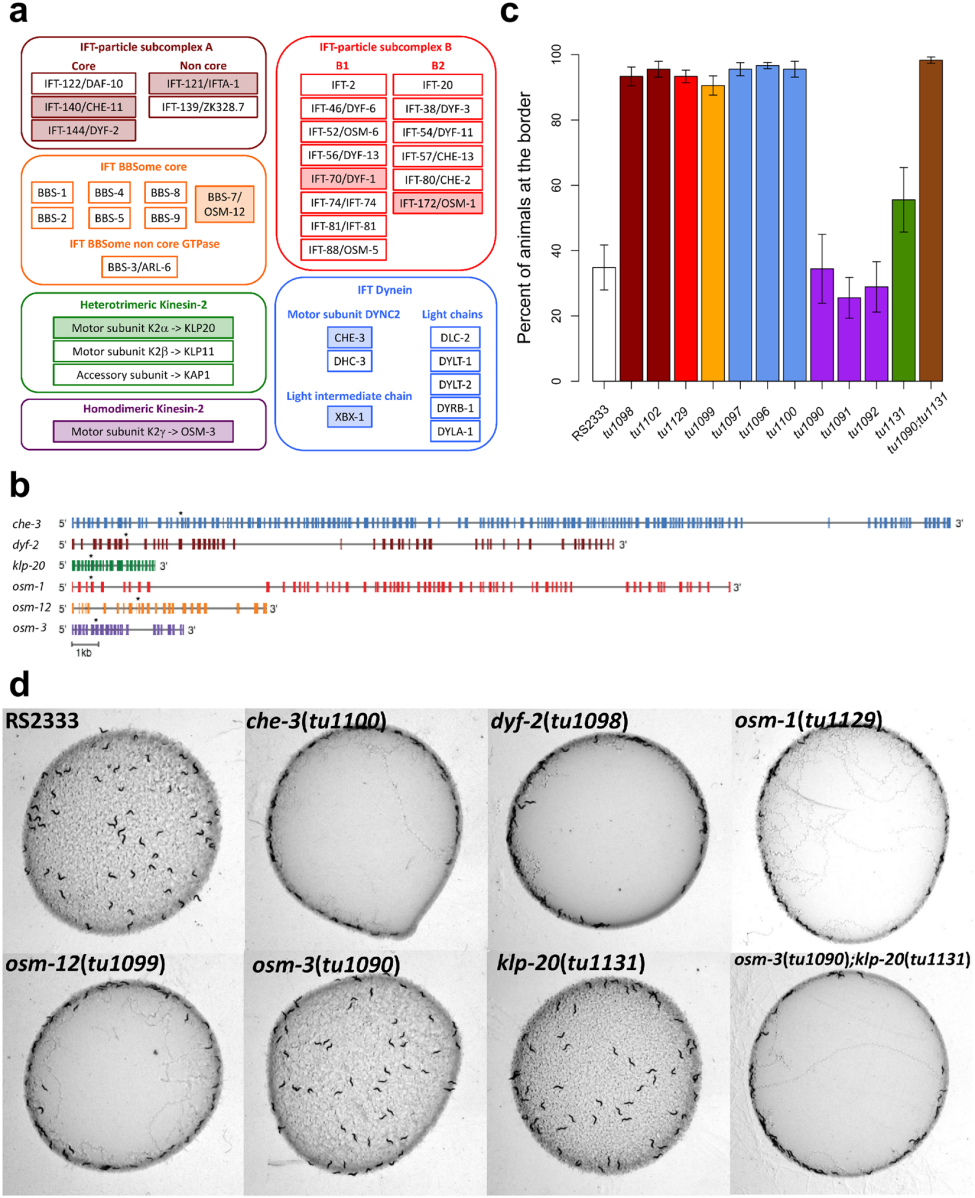
Social behaviours induced by mutations in other genes encoding intraflagellar transport proteins. **(a)** Orthologs of IFT-related genes in *P. pacificus*. Colour-filled boxes indicate genes for which mutant alleles have been produced using CRISPR/Cas9 system in this study. **(b)** Gene structure of the *Ppa-che-3*, *Ppa-dyf-2*, *Ppa-osm-1*, *Ppa-osm-3*, *Ppa-osm-12* and *Ppa-klp-20* genes. Colour boxes indicate exons and solid lines indicate introns. Asterisks indicate sgRNA positions. **(c)** Bordering behaviour of RS2333, mutant alleles of *Ppa-che-3*, *Ppa-dyf-2*, *Ppa-osm-1*, *Ppa-osm-3*, *Ppa-osm-12* and *Ppa-klp-20* produced using CRISPR/Cas9 system (Suppl. Table S8), plus *Ppa-osm-3*(*tu1090*)*; Ppa-klp-20*(*tu1131*) double mutant. Colours correspond to genes in Fig. 4b and functional groups in Fig. 4a. In all bar-plots arrows represent the standard error of the mean (SEM). For statistical analysis see Suppl. Table S9. **(d)** Clumping/bordering assay for representatives of *Ppa-che-3*, *Ppa-dyf-2*, *Ppa-osm-1*, *Ppa-osm-3*, *Ppa-osm-12* and *Ppa-klp-20* mutant alleles and the *Ppa-osm-3*(*tu1090*)*; Ppa-klp-20*(*tu1131*) double mutant.

IFT requires the coordinated action of six multi-protein sub-complexes: the IFT-sub-complex A and B, the BBSome, the homodimeric and heterotrimeric kinesin motors and the dynein motors^22,23^. To test whether other IFT proteins are also involved in the inhibition of social behaviours in RS2333, we identified the *P. pacificus* orthologs of all IFT related genes (see methods) (Fig. 4a). Next, we employed a candidate gene approach by selecting one representative of each of the six multi-protein sub-complexes to produce knock-out mutants using the CRISPR/Cas9 system: *dyf-2* and *osm-1* from the IFT-A and IFT-B sub-complexes, *osm-12* from the BBSome, the heavy chain *che-3* component of the IFT dynein motor, the *klp-20* component from the heterotrimeric kinesin-II motor and the homodimeric OSM-3-kinesin motor (Fig. 4b and Suppl. Fig. S3).

We were able to isolate between one and three alleles per targeted gene (Suppl. Table S8). Mutants in *Ppa*-*dyf-2*, *Ppa-osm-1*, *Ppa-osm-12* and *Ppa-che-3* showed social phenotypes (Fig. 4c,d and Suppl. Table S9), indicating that proteins of the IFT-A, IFT-B, the BBSome and the IFT dynein motor sub-complexes are indeed involved in the inhibition of social feeding in *P. pacificus* RS2333. These findings also indicate that our original mutagenesis screen was far from saturation.

In contrast to the CRISPR/Cas9-induced mutants in *Ppa-dyf-2*, *Ppa-osm-1*, *Ppa-osm-12* and *Ppa-che-3*, mutants of each kinesin motor holoenzymes remained solitary. Specifically, all three alleles in *Ppa-osm-3* and the single *Ppa-klp-20*(*tu1131*) mutant showed a normal solitary phenotype (Fig. 4c,d and Suppl. Table S9). Two possible scenarios might explain these findings; either, kinesin motors are not involved in the inhibition of the social behaviours, or alternatively, there might be functional redundancy between the heterotrimeric kinesin-II and the homodimeric OSM-3-kinesin motors as previously described for *C. elegans* ^27^. To distinguish between these scenarios, we generated a kinesin motor double mutant and indeed, *Ppa-osm-3*(*tu1090*); *Ppa-klp-20*(*tu1131*) double mutant animals showed strong social behaviours (Fig. 4c,d and Suppl. Table S9). These findings suggest that kinesin motors are involved in the inhibition of social behaviours in the wild type RS2333 strains and that the kinesin motors function redundantly in *P. pacificus*.

### IFT mutants in *P. pacificus* are defective for chemosensation and dye-filling of amphid neurons

**Figure 5:**
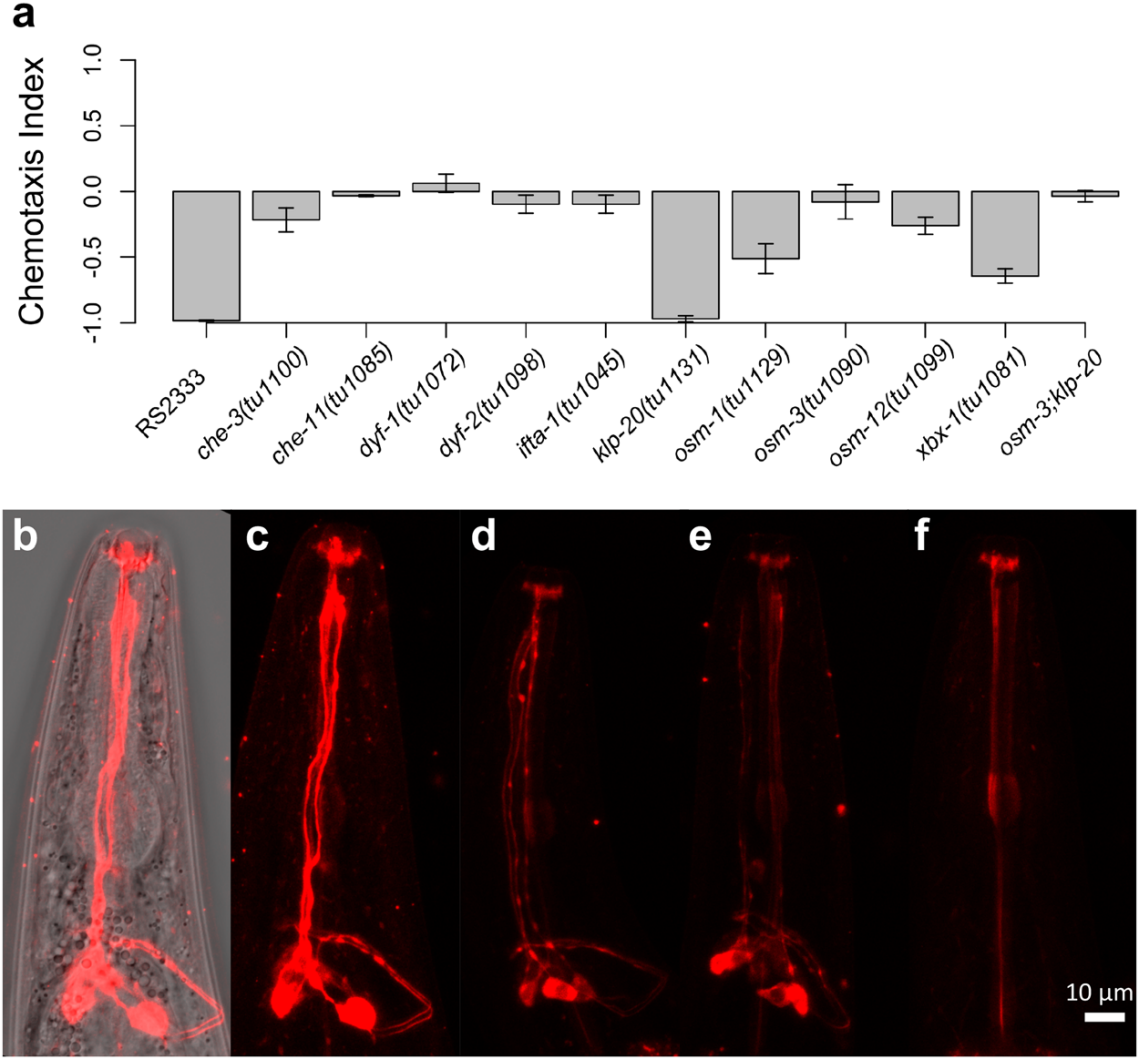
Chemosensation and dye-filling defects in *P. pacificus* IFT mutants. **(a)** 1-Octanol avoidance behaviour of RS2333 and the IFT-related mutant alleles generated in this study. For statistical analysis see Suppl. Table S10. (**b)** Overlay of bright field and fluorescent images showing dye-filling staining of amphid neurons in a RS2333 adult individual. (**c)** Dye-filling staining of amphid neurons in a RS2333 adult. **(d)** Dye-filling staining of amphid neurons in an *osm-3*(*tu1090*) adult. (**e)** Dye-filling staining of amphid neurons in a *Ppa-klp-20*(*tu1131*) adult. (**f)** Dye-filling defective staining of amphid neurons in an *Ppa-osm-3*(*tu1090*); *Ppa-klp-20*(*tu1131*) double mutant.

In *C. elegans* IFT mutants were characterized by performing dye-filling assays for the staining of ciliated neurons, chemotaxis and osmotic avoidance behaviours^28^. To study whether IFT mutants in *P. pacificus* show abnormal chemotaxis behaviour we assayed repulsion from 1-Octanol, a chemical known to induce strong avoidance in *P. pacificus* wild type animals^29^ (Fig. 5a and Suppl. Table S10). Indeed, the chemotaxis index was close to 0 for most of the IFT mutants, indicating that they are unable to sense 1-Octanol. However, *Ppa-osm-1*(*tu1129*) and *Ppa-xbx-1*(*tu1081*) mutants showed only a reduction in the avoidance behaviour, while *Ppa-klp-20*(*tu1131*) mutants appeared wild type. The difference in the repulsion behaviour between *Ppa-osm-3*(*tu1090*) and *Ppa-klp-20*(*tu1131*) mutants could indicate that the receptor triggering this behaviour is located at the distal segment of the cilia, since in *C. elegans* only the homodimeric OSM-3-kinesin motor carries the IFT particles to the distal cilia tip^27^.

Absence of aversion by 1-Octanol in IFT mutants in addition to the previously described social phenotype supports the conserved function of the studied IFT proteins in ciliogenesis. To detect crude morphological changes in ciliated neurons in IFT mutants, we used a dye-filling technique, whereby nematodes were incubated with the lipophilic dye DiI, which enters amphids and other sensory neurons if they are fully formed and open to the outside^30^. In the wild-type RS2333 strain, we observed intense staining of the neurons corresponding to amphid neurons in *C. elegans* also known to be stained by DiI (Fig. 5b,c). Strikingly, staining was absent in most of the mutants examined. The only exceptions were *Ppa-osm-3*(*tu1090*) and *Ppa-klp-20*(*tu1131*) (Fig. 5d,e), however, the double mutant was also dye-filling defective, similar to all of the other IFT mutants (Fig. 5f). The absence of dye-filling is consistent with the expectation of abnormal morphogenesis of ciliated neurons regulated by IFT genes. This conclusion is further supported by strong correlation of the dye-filling pattern and the social phenotypes of the mutant strains.

### *P. pacificus* IFT mutants modulate clumping and bordering and locomotive behaviours in response to environmental oxygen levels

**Figure 6:**
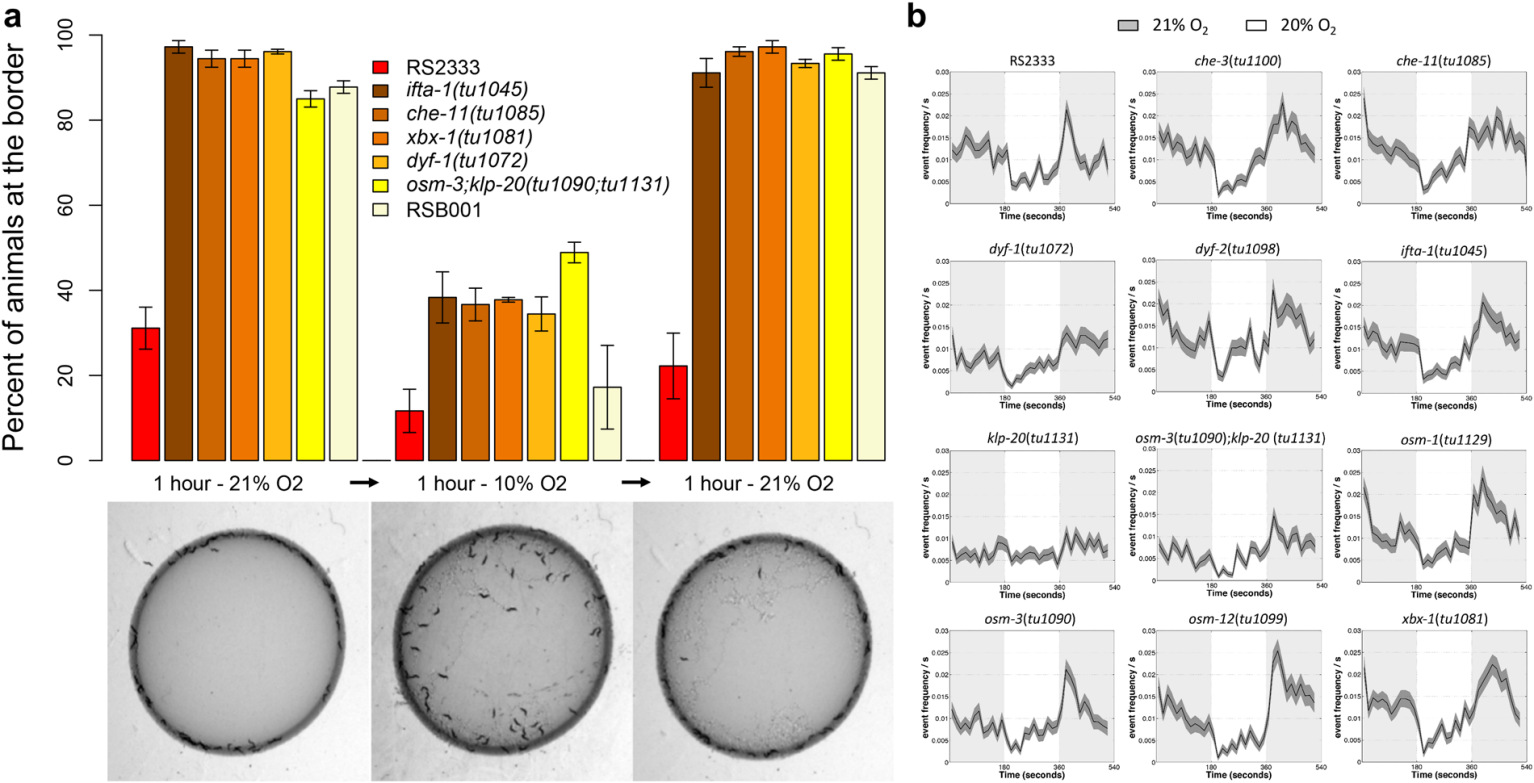
Hyperoxia avoidance behaviours in *P. pacificus* IFT mutants. **(a)** Regulation of bordering behaviour by oxygen levels in RS2333, RSB001 and representatives of *Ppa-ifta-1*, *Ppa-che-11*, *Ppa-dyf-1* and *Ppa-xbx-1* mutants. [O_2_] shifts every hour from 21% to 10% to 21%. Three replicates were performed for each strain/mutant. Bar-plots arrows represent the standard error of the mean (SEM). For statistical analysis see Suppl. Table S11. An example of the influence of oxygen in the dynamic of clumping/bordering behaviours for *tu1072* is shown under the bar-plot. (**b)** Ω-turn rate response to 21% −>20% −>21% [O_2_] shifts on a lawn of *E. coli* OP50 of RS2333 and all cilia-related mutants generated using CRISPR/Cas9 system in this study. In all graphs, the black line represents the mean Ω-turn and the grey area, the S.E.M. For statistical analysis see Suppl. Table S12.

Finally, we tested whether the social behaviours of IFT mutants were still regulated by [O_2_] using an aerotaxis chamber (see methods). In all mutant lines tested, the social behaviour showed the following dynamics: 10% [O_2_] induced a strong inhibition of bordering from 90–100% to 35–40%, while the increase to 21% [O_2_] induced bordering close to 100% (Fig. 6a, Suppl. Table S11a,b). These findings indicate that the social behaviour observed in the IFT mutants are established with the intent to avoid hyperoxic conditions, since a significant decrease in the [O_2_] produced a strong reduction in bordering. Since these mutant lines are most probably defective in sensory-cilia related processes, our experiments suggest that environmental inputs sensed through cilia are required in the solitary *P. pacificus* RS2333 strain to counteract hyperoxic stress conditions, resulting in the inhibition of clumping and bordering. In addition, the bordering behaviour of RS2333 fluctuated from 20–30% at 21% [O_2_] to 12% at 10% [O_2_]. This reduction is equivalent in magnitude to what we observed in the IFT mutants, which suggests that [O_2_] affects both wild-type and IFT mutant strains equivalently (Fig. 6a and Suppl. Table S11a,b), likely indicating parallel regulatory inputs consisting of the low [O_2_] and cilia-mediated mechanism contributing to the inhibition of social behaviours in RS2333.

Furthermore, we tested the ability of the mutants to respond to small shifts from 20% to 21% [O_2_] by increasing their rate of Ω-turns. We observed that all IFT mutants were responsive to [O_2_] as predicted (Fig. 6b and Suppl. Table S12), although the rate of Ω-turns varies among them. Specifically, for the *Ppa-dyf-1*(*tu1072*), *Ppa-klp-20*(*tu1131*) and the *Ppa-osm-3; Ppa-klp-20* double mutants the difference in the Ω-turn rate was lower in comparison to wild type (Fig. 6b and Suppl. Table S12). These findings indicate that both the *klp-20* and *dyf-1* genes may play a role in the regulation of the Ω-turn rate in response to shifts in [O_2_]. However, the reduction of the locomotive response in these mutants seems not to affect the regulation of its social behaviour by [O_2_]. Indeed, the social behavior of the *Ppa-osm-3; Ppa-klp-20* double mutant was strongly suppressed by hypoxia (Fig. 6a, Suppl. Table S11a,b), in spite of its weak Ω-turn response to [O_2_] shifts.

## Discussion

Social behaviour in animals is frequently employed for stress avoidance and also as a means of defence. Examples of social cooperation to build shelters are found among both vertebrates and invertebrates, e.g. beehives, rodent burrows and termite nests^31^. In addition, living in groups offers passive or active protection from predators as passive collective defence involves surrounding themselves with others^32^, while active involves adopting radial defensive formation, which has been observed among insects^33^ and vertebrates^34^. Aggregation is likewise employed to gain protection from environmental stress, especially in thermoregulation, which has been observed among mammals, reptiles and insects^35–37^.

Among nematodes, aggregation is addressed to avoid another kind of stress: hyperoxia. Wild type isolates of *C. elegans* perform a clumping behaviour under laboratory conditions in order to escape from the atmospheric 21% [O_2_]^3,5^. In addition, they aggregate at the border of the bacterial lawn, which is thicker and consumes more O_2_ than the centre, to further reduce the [O_2_] to which they are exposed^4^. This hyperoxia-avoidance behaviour is probably related to the adaptation of *C. elegans* to the hypoxic natural habitats where it is usually found, i.e. compost heaps and rotten fruits^6^. In these environments avoidance of 21% [O_2_] would be beneficial in order to escape desiccation by surface exposure, while additionally enabling their accumulation on bacterial food sources^38^. On the contrary, most of the *P. pacificus* nematode wild isolates show a solitary foraging behaviour under laboratory conditions, which indicates that unlike *C. elegans*, solitary foraging in *P. pacificus* is ancestral, and it does not constitute a lab-derived artefact related to domestication^15^.

**Figure 7:**
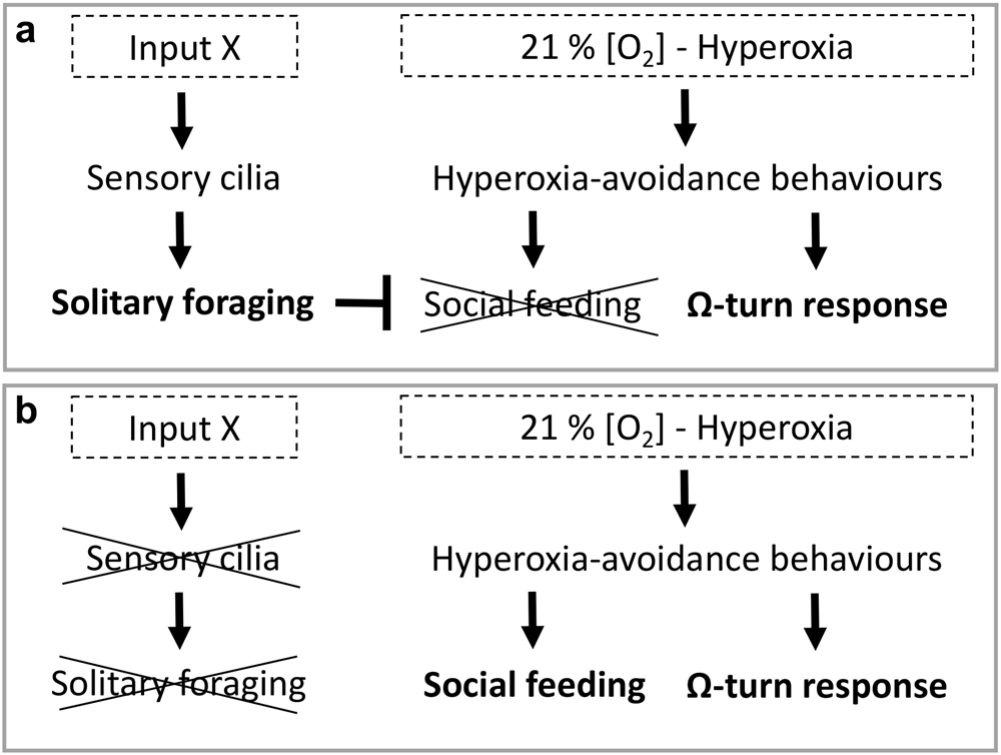
Model for the regulation of hyperoxia-avoidance behaviours (social feeding and Ω-turn rate) by oxygen and the input integrated through sensory-cilia. (**a)** In wild type solitary strains, hyperoxic conditions increase the Ω-turn rate while crawling. However, the hyperoxia-avoidance by social behaviour is suppressed by the input X, which is recognised by sensory cilia. (**b)** In cilia-defective mutants the input X does not inhibit hyperoxia-avoidance by social behaviour in response to hyperoxic conditions.

Surprisingly *P. pacificus* solitary strains show a strong Ω-turn response to shifts from 20% to 21% in [O_2_], which indicates that these strains are able to perceive 21% [O_2_] as hyperoxic stress, but nonetheless, they do not try to escape the hyperoxic stress conditions by performing clumping and bordering. In this work, we have demonstrated the existence of a novel regulatory mechanism in the solitary strain RS2333, which blocks the social behaviours even under hyperoxic stress conditions. By means of both forward and reverse mutagenesis, we have produced social mutants from RS2333, which are defective in IFT genes, essential for the proper assembly of the cilia and its function in sensing and signalling^24^. Therefore, these experiments provide strong evidence for the existence of a regulatory pathway triggered by an environmental signal, which must be recognised by ciliated neurons and in turn leads to the inhibition of the social behaviours (Fig. 7a). On the contrary, in IFT defective mutants this regulatory pathway acting though the cilia is impeded, and as a consequence, the inhibition of social behaviours is abrogated allowing the nematodes to escape hyperoxic stress conditions by means of clumping and bordering (Fig. 7b).

Our current hypothesis to explain the evolution of this mechanism proposes that it arose as a consequence of the ecological association of *P. pacificus* with beetle hosts. While in *C. elegans* social feeding may be advantageous in the wild in order to remain in contact with patches of bacterial food sources, the need for *P. pacificus* nematodes developing on the carcass of the previous beetle host to find a new host may have favoured the emergence of a mechanism that induces solitary foraging and ultimately favours dispersion. However, the nature of the environmental cue that triggers the solitary foraging behaviour remains unknown and it will be explored in future studies. Our observation of a strong correlation between social behaviours and dye-filling staining in amphid neurons in the IFT defective *P. pacificus* mutants, indicates that this putative environmental trigger is probably sensed by the amphid neurons. Future work will take advantage of the well-known synaptic connectivity of *P. pacificus* ^39^ to analyse the role of the amphid neurons in this inhibitory mechanism by neural ablation or induced apoptosis by reporter system methodologies.

In *C. elegans*, IFT defective mutants show abnormal chemotaxis behaviours as well as morphological defects in cilia^28^. Yet, to our knowledge, social behaviours have not been reported in *C. elegans* IFT defective mutants, indicating that the inhibitory mechanism of the social behaviours suggested for *P. pacificus* is absent in *C. elegans*. However, it has been reported that noxious stimuli from bacterial food sources sensed through the ASH and ADL amphid neurons induce aggregation in *C. elegans npr-1*(*null*) mutants. This mechanism requires the TRP-related transduction channels *ocr-2* and *osm-9*, as well as *odr-4* and *odr-8*, which localise sensory chemoreceptors to cilia^40^. Specifically, the solitary behaviour of *npr-1; ocr-2* and *npr-1; odr-4* double mutants was suppressed in triple mutants including *osm-3*, indicating that inputs from neurons that express *osm-3* inhibit aggregation^40^. This inhibitory mechanism is fundamentally different from the one described here for *P. pacificus* since the latter constitutes the main mechanism suppressing clumping and bordering in natural strains while the former was evident only when *osm-3* plus *ocr-2* or *odr-4* were knocked-out on a *npr-1*(*null*) background, whereas *osm-3* single mutants show no social phenotype.

In conclusion, our study reveals a new additional level of complexity in the regulation of the hyperoxia-induced social behaviours in *P. pacificus* in comparison with *C. elegans*. In addition, it demonstrates the need to work on animal models with a well-known ecology in order to disentangle biological questions related to evolution and adaptation in the wild.

## Methods

### Strains

Three *P. pacificus* strains were used in this study: the reference strain PS312 (belonging to the phylogenetic lineage A and isolated from Pasadena, CA (USA) in 1988^13^), the RS2333 strain (a laboratory derivative of the original strain PS312) and the RSB001 strain (belonging to the phylogenetic lineage B and isolated from a location at 2327 m.a.s.l. on La Réunion Island^15,16^). Strains were maintained at 20 °C using standard methods^41^.

### Behavioural Assays

The assay for quantification of bordering and clumping behaviours in *C. elegans* ^3^ was modified for *P. pacificus* as previously indicated^15^. The regulation of bordering behaviour by oxygen was analysed by performing the above assay in a custom-fabricated Plexiglas chamber^20^ as previously indicated^15^ with three replicates completed per strain/mutant. Nematodes where exposed to shifting [O_2_] levels from 21% to 10% and back to 21% after one hour intervals. Oxygen-evoked turning responses were monitored as described previously^15,20,42,43^ with ten replicates completed per strain/mutant. Oxygen concentration was changed every three min. Ω-turn rate values were calculated in 15 sec windows. The means of Ω-turns during the 90 sec before and after the oxygen shift were calculated for each replicate. The chemotaxis assay to quantify 1-Octanol avoidance behaviour was modified from Hong *et al*.^44^ as follows: For each strain/mutant, nematode cultures were synchronized by transferring ten gravid hermaphrodites onto 6-cm NGM plates seeded with *Escherichia coli* OP50. Ten plates were prepared per strain/mutant, which were then incubated at 20 °C for five days. Nematodes were then washed with M9 buffer and filtered with a 5 µm nylon membrane filter (Merck Millipore, Billerica, MA USA) to remove the bacteria. The animals were loaded onto 8.5-cm NGM plates between the test compound (1 µl of 100% 1-Octanol (Sigma-Aldrich Co., St. Louis, MO USA)) and the control compound (1 µl of 100% Ethanol (Merck Millipore)), located at opposed ends of the plates. Plates were incubated at 20 °C for 12 hours. Animals within 2 cm radius-circle around each odour source were counted. Three replicates for each strain/mutant were carried out and the differences in chemotaxis index between the reference strain RS2333 and each mutant allele were calculated as in Hong *et al*.^44^ by means of the two-sample equal variance Student’s t test.

### Mutant screen

We screened for bordering/clumping mutant strains using Ethyl methanesulfonate (EMS) mutagenesis^41^ in *P. pacificus* RS2333. We screened approximately 2,250 gametes (4,500 homozygous F_2_ lines) in three mutagenic screens over a six-month period. The screens were performed by placing single F2s in a 50 µl OP50 lawn. Then the bordering behaviour was screened on the F3 animals. Candidate mutants were confirmed by proper bordering/clumping assays as described before.

### CRISPR/Cas9 mutagenesis

*P. pacificus* strains PS312, RS2333 and RSB001 were used for generating mutants using CRISPR/Cas9 system^45^ following the protocol of Witte *et al*.^19^. For most of the genes, sgRNAs were designed to target sequence regions conserved between *P. pacificus* and *C. elegans*, as identified in amino acid sequence alignments produced using Mafft version 7^46^. sgRNAs were synthesized by ToolGen Inc. (Seoul, Korea) and Integrated DNA Technologies Inc. (Coralville, Iowa USA). We used Cas9 protein produced by ToolGen Inc. and New England BioLabs Inc. (Ipswich, MA USA). Injection Master Mix was prepared following the manufacturer instructions from Alt-R^TM^ CRISPR-Cas9 System User guide from Integrated DNA Technologies Inc. Injections were performed on a Zeiss Axiovert microscope (Zeiss, Germany) coupled to an Eppendorf TransferMan micromanipualtor and Eppendorf FemtoJet injector (Eppendorf AG., Hamburg, Germany). Injected mothers were kept individually on NGM plates for 16 hours. Around 100 F1 progeny were transferred onto NGM plates before they became adults (one individual per plate). Once they have laid eggs, F1 individuals were lysed and assayed for the presence of a molecular lesion around the sgRNA target site. For *npr-1* and *ifta-1* genes this was performed by PCR and subsequent Sanger sequencing. For the rest of the genes, high resolution melting was performed using LightCycler 480 High Resolution Melting Master (Roche Diagnostics Ltd., Burgess Hill, England) and the identified mutant candidates were confirmed by Sanger sequencing. Primers for detecting gene lesions are listed in Suppl. Table S13.

### Genetics

To analyse the dominant/recessive character of social feeding behaviours of the mutant alleles, we performed the bordering/clumping assays on heterozygotes made by crossing males of each mutant strain, with hermaphrodites of the dumpy (*pdl-1*) mutant version of RS2333. After backcrossed with the RS2333 *pdl-1* mutant, mutant strains showing both social and dumpy phenotypes were isolated for further complementation tests, which were performed by crossing males of each mutant strain, with hermaphrodites of each backcrossed mutant strain.

### Whole genome re-sequencing of mutant strains

Genomic DNA was prepared for all mutant strains following the protocol outlined in Rödelsperger *et al*.^47^. DNA was extracted from pooled individuals of each isogenic line using the GenElute^TM^ Mammalian Genomic DNA Miniprep Kits (Sigma-Aldrich Co., St. Louis, MO USA) and genomic DNAs were quantified with the Qubit® dsDNA BR Assay Kit (Thermo Fisher Scientific Inc., Waltham, MA USA). Genomic libraries were generated using the TruSeq Nano DNA Library Prep Kit from Illumina (Illumina Inc., California, United States). DNA was sheared to 350 bp using the Covaris S2 System (Covaris Ltd., Woodingdean Brighton, United Kingdom) and end repair, adenylation, and adaptor ligation were performed following the kit protocol. After PCR amplification, libraries were validated on an Agilent Bioanalyzer DNA 1,000 chip (Agilent Technologies GmbH, Waldbronn, Germany) and pooled before sequencing on an Illumina HiSeq. 3000 platform.

### Identification of mutant alleles from whole-genome sequencing data

Raw Illumina reads were aligned to the *P. pacificus* genome (version Hybrid1) and candidate variants were called, filtered, and classified as described in Rae *et al*.^48^. This yielded between 400–600 substitutions per mutant line, for which roughly 10% were located in coding regions and were predicted to affect the gene product (non-synonymous, nonsense, or splice-site mutations). To identify complementation groups, we intersected the lists of affected genes between different mutant samples.

### Orthologs Gene Finding

*P. pacificus* orthologs for 31 out of 39 cilia-related *C. elegans* genes were obtained from automated orthology predictions by Baskaran *et al*.^49^. The orthologs for the remaining eight genes were identified by manual BLAST analysis against different *P. pacificus* databases (genome, gene annotations, and trancriptomes) on www.pristionchus.org.

### Dye-filling

The dye-filling protocol was adapted from previously described methods^50^. Nematodes were transferred into 1.5 ml centrifuge tubes and prewashed 3 times with M9 medium. Washed animals were stained with Vybrant DiI Cell-Labelling Solution (Thermo Fisher Scientific Inc.) diluted 100-fold in 250 µl of M9 medium. After incubation in a rocking wheel for 3 h, nematodes were washed 3 times with M9 medium to remove excess dye. Adult individuals were picked to agar pads containing 0.3% w/v NaN_3_ and imaged using a Leica TCS SP8 confocal microscope. Identical acquisition settings were used for all images. Average intensity Z-projections and overlay of bright field and fluorescent images were created using FIJI software^51^. Intensity ranges displayed in the fluorescent images in Fig. 4 are identical.

### Statistical analyses

Statistical analyses were performed in the computing environment R ver. 3.1.3 (R Core Team, 2015). Replicates of clumping/bordering assays, aerotaxis assays and chemotaxis assays were used to calculate means and standard errors (S.E.M.). Two-sample equal variance Student’s t test, with Bonferroni corrections for multiple hypothesis testing, was used to confirm significant differences in bordering averages between wild type and mutant strains, Ω-turn rate averages before and after oxygen shifts, and chemotaxis index between wild type and mutant strains.

## Electronic supplementary material

Supplementary information
