# Supplementary material for: Regulation of hyperoxia-induced social behaviour in Pristionchus pacificus nematodes requires a novel cilia-mediated environmental input
Source: Sci Rep. 2017 Dec 14;7:17550. doi: 10.1038/s41598-017-18019-0 (PMC5730589; doi:10.1038/s41598-017-18019-0)
Supplement: Supplementary file 1 — Supplementary information [file 41598_2017_18019_MOESM1_ESM.pdf]

Regulation of hyperoxia-induced social behaviour in *Pristionchus pacificus* nematodes requires a novel cilia-mediated environmental input

Eduardo Moreno[a1], Bogdan Sieriebriennikov[a], Hanh Witte[a], Christian Rödelsperger[a], James W. Lightfoot[a] & Ralf J. Sommer [a1].

[a] Department for Integrative Evolutionary Biology, Max Planck Institute for Developmental Biology, 72076 Tübingen, Germany.

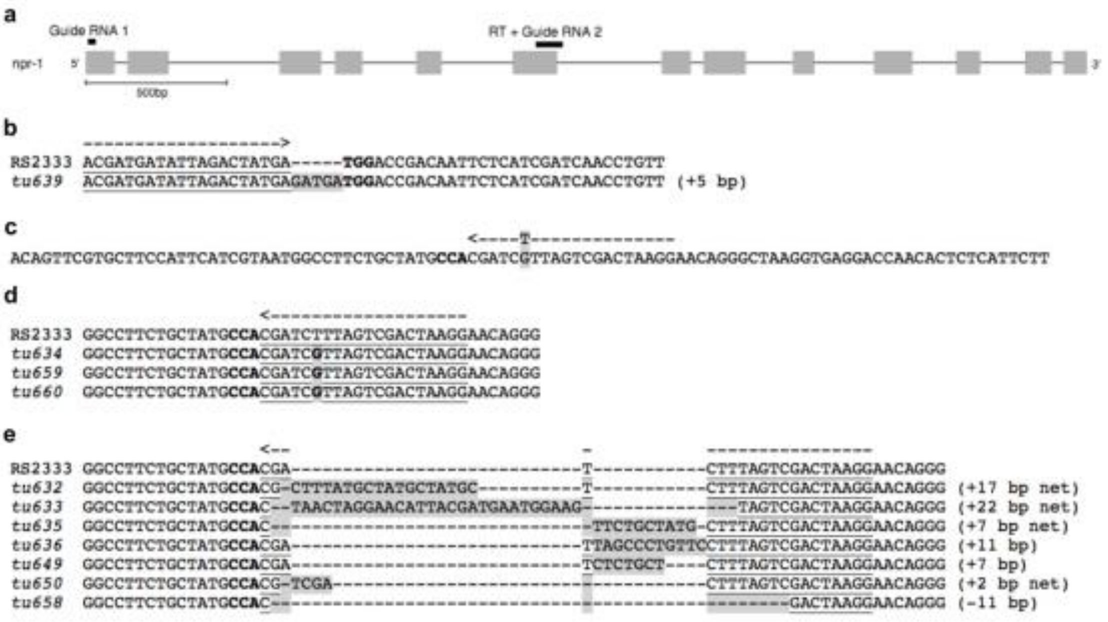

Supplementary Figure S1. *Ppa-npr-1* mutant alleles produced using CRISPR/Cas9 system. (**a**) Gene structure of the *npr-1* ortholog in *P. pacificus*. Grey boxes indicate exons and solid lines indicate introns. The positions of the sgRNA sequences and the repair-template oligonucleotide (RT) are indicated. (**b**) Genetic lesions in the *Ppa-npr-1* mutant allele *tu639*, generated in exon 1. (**c**) Repair template oligonucleotide containing a single nucleotide substitution (T->G), which induces the corresponding amino-acid substitution F->V. (**d**) *npr-1* gain-of-function mutant alleles. (**e**) Genetic lesions in *Ppa-npr-1* mutant alleles generated in exon 6. In all figures arrows indicate sgRNA sequences, the PAM domain is shown in bold and the induced genetic lesions are highlighted in grey colour.

ifta-1

```
RS2333 GATTCTCGACGATGACAGCAACA     CACTGGCCTGCT
tu1044 GATTCTCGACGATGACAGCAACATCGTACACTGGCCTGCT (+5 bp)
tu1045 GATTCTCGACGATGACAGC-------------TGGCCTGCT (-7 bp)

RS2333 CCGCTTCCCCAACGT-------CACCCTCACGTCGGCCCAGTGG
tu1123 CCGCTTCCCCAACGTGGACCAA---------------CCAGTGG (+8 bp net)
```

dyf-1

```
RS2333 GACGGAGAGTACACAAGTACAATCTATGGATTGGTGAGATTCTTTTAACCATATTTT
tu1072 GACGGAGAGTACACAAGTACA-----TGGATTGGTGAGATTCTTTTAACCATATTTT (-5 bp)
tu1074 GACGGAGAGTACACAAGTACAACC-----------------------ACCATATTTT (+23 bp net)
```

che-11

```
RS2333 GAAGCAACAGATAACAGTGCCTG-----------AGATGGAAGGAG
tu1085 GAAGCAACAGATA----------TATAACAGATAAGATGGAAGGAG (+1 bp net)
tu1086 GAAGCAACAGATA----------GA---------AGATGGAAGGAG (-8 bp net)
tu1087 GAAGCAACAGATAACAGTGCCTGATGGA------AGATGGAAGGAG (+5 bp)
```

xbx-1

```
RS2333 TTCAGACTAAGCAACTGTGTCACGTGTGGGAGTTG
tu1085 TTCAGACTAAGCAACTGTGTCGAG---GGGAGTTG (+3 bp net)
```

Supplementary Figure S2. Mutant alleles of *Ppa-ifta-1*, *Ppa-dyf-1, Ppa-che-11* and *Ppa-xbx-1* produced using CRISPR/Cas9 system. Arrows indicate sgRNA sequences, the PAM domain is shown in bold and the induced genetic lesions are highlighted in grey colour.

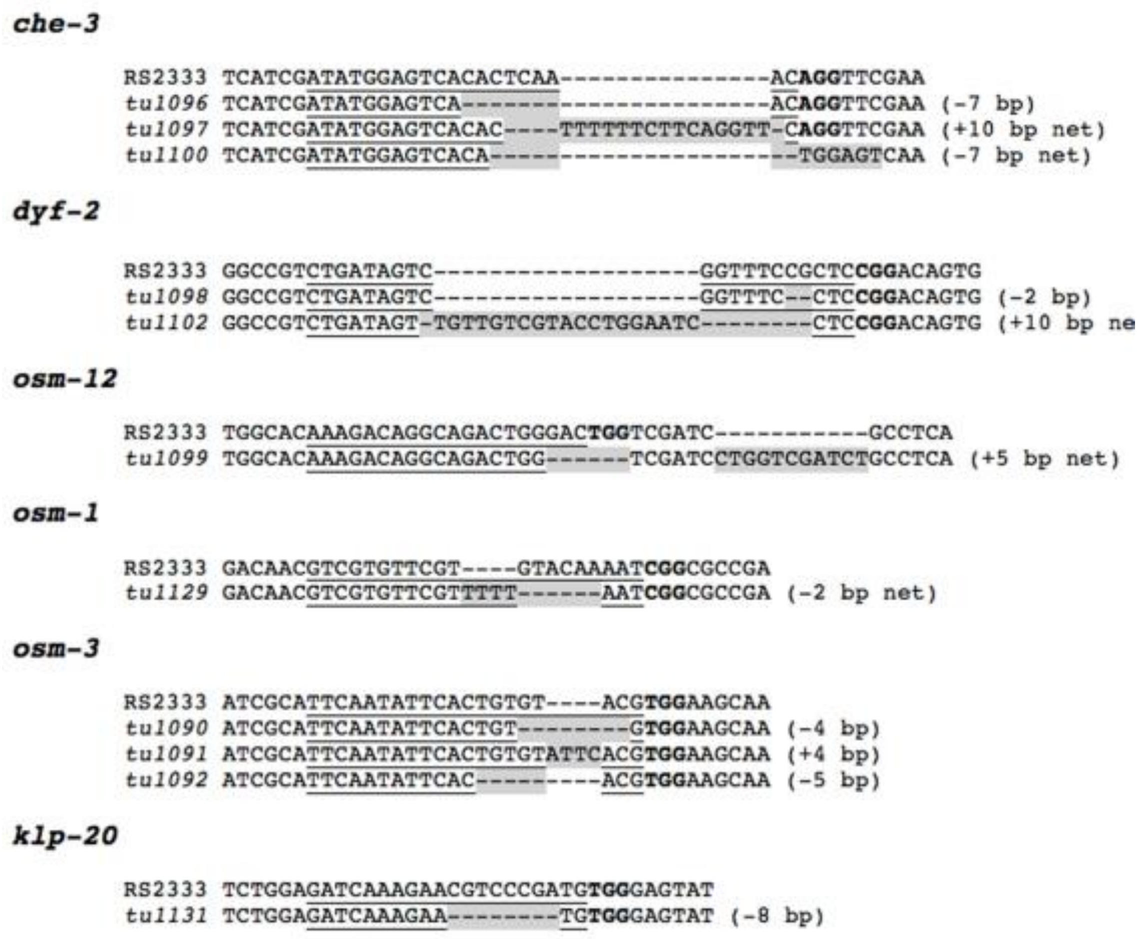

Supplementary Figure S3. Mutant alleles of *Ppa-che-3*, *Ppa-dyf-2*, *Ppa-osm-1*, *Ppa-osm-3*, *Ppa-osm-12* and *Ppa-klp-20* produced using CRISPR/Cas9 system. Arrows indicate sgRNA sequences, the PAM domain is shown in bold and the induced genetic lesions are highlighted in grey colour.

Allele	Mutation	Type of mutation	Strain
tu634	SNP : F->V	gain-of-function	PS312
tu632	1 bp deletion + 18 bp insertion	nonsense	PS312
tu633	6 bp deletion + 28 bp insertion	nonsense	PS312
tu635	3 bp deletion + 10 bp insertion	nonsense	PS312
tu636	11 bp insertion	nonsense	PS312
tu639	5 bp insertion	nonsense	PS312
tu659	SNP : F->V	gain-of-function	RSB001
tu660	SNP : F->V	gain-of-function	RSB001
tu649	7 bp insertion	nonsense	RSB001
tu650	2 bp deletion + 4 bp insertion	nonsense	RSB001
tu658	11 bp deletion	nonsense	RSB001

Supplementary Table S1. Mutant alleles of the *npr-1* ortholog in *P. pacificus* produced using CRISPR/Cas9 system. For each allele, the length of the genetic lesion, the type of mutation and the background strain from which the mutant was produced are indicated.

x	y	mean x	mean y	mean difference	t	df	*P*-value[1]	significance
RS2333	*tu634*	34.83	23.83	11	1.61	18	0.124	ns
RS2333	*tu632*	34.83	49.16	14.32	-2.30	18	0.033	ns
RS2333	*tu633*	34.83	53.00	18.17	-2.73	18	0.014	ns
RS2333	*tu635*	34.83	59.67	24.84	-4.13	18	< 0.001	**
RS2333	*tu636*	34.83	49.66	14.18	-2.39	18	0.028	ns
RS2333	*tu639*	34.83	54.5	19.67	-3.21	18	0.005	ns
RSB001	*tu659*	86.83	83.00	3.83	1.50	18	0.150	ns
RSB001	*tu660*	86.83	85.17	1.66	0.67	18	0.513	ns
RSB001	*tu649*	86.83	97.5	10.67	-4.64	18	< 0.001	**
RSB001	*tu650*	86.83	97.16	10.33	-4.35	18	< 0.001	**
RSB001	*tu658*	86.83	95.67	8.84	-3.60	18	0.002	*

Supplementary Table S2. Statistical values from pairwise comparisons for mean bordering behaviour between wild-type strains and *Ppa-npr-1* CRISPR mutants. Each strain/mutant was measured 10 times. The percentage means of bordering behaviour were compared using Student's t-test.

[1]The alpha value, corrected for multiple comparisons via the Bonferroni method, corresponds to 0.05/11 = 0.004.

Strain	mean 20% [O_2]	mean 21% [O_2]	mean difference	t	df	*P*-value[1]	significance
RS5412	0.003	0.014	0.011	-5.02	10	< 0.001	**
RS5399	0.008	0.019	0.011	-5.73	10	< 0.001	***
RSA047	0.007	0.019	0.012	-5.17	10	< 0.001	**

Supplementary Table S3. Ω-turn rates in response to oxygen concentration ([O_2]) shifts from 20% to 21% on food in selected *P. pacificus* solitary strains. The statistical values from pairwise comparisons between mean Ω-turns before and after oxygen shifts were calculated with the Student's t-test.

[1] The alpha value, corrected for multiple comparisons via the Bonferroni method, corresponds to 0.05/3 = 0.0167

x	y	mean x	mean y	mean difference	t	df	*P*-value[1]	significance
RS2333	*tu662*	34.83	87.50	52.67	-9.03	18	< 0.001	***
RS2333	*tu663*	34.83	83.17	48.34	-7.49	18	< 0.001	***
RS2333	*tu664*	34.83	96.67	61.84	-10.78	18	< 0.001	***
RS2333	*tu665*	34.83	91.67	56.84	-9.51	18	< 0.001	***
RS2333	*tu666*	34.83	94.16	59.33	-10.10	18	< 0.001	***
RS2333	*tu667*	34.83	96.83	62.00	-10.82	18	< 0.001	***
RS2333	*tu668*	34.83	94.66	59.83	-10.24	18	< 0.001	***
RS2333	*tu670*	34.83	96.83	62.00	-10.75	18	< 0.001	***
RS2333	*tu671*	34.83	95.00	60.17	-10.15	18	< 0.001	***
RS2333	*tu672*	34.83	92.67	57.84	-9.73	18	< 0.001	***
RS2333	*tu674*	34.83	79.67	44.84	-6.50	18	< 0.001	***
RS2333	*tu675*	34.83	90.33	55.50	-9.51	18	< 0.001	***
RS2333	*tu676*	34.83	82.33	47.50	-7.24	18	< 0.001	***
RS2333	*tu678*	34.83	90.83	56.00	-9.47	18	< 0.001	***
RS2333	*tu669*	34.83	93.83	59.00	-10.10	18	< 0.001	***

Supplementary Table S4. Statistical values from pairwise comparisons for mean bordering between RS2333 and the 15 social mutants isolated in chemical mutagenesis screens. Each strain/mutant was measured 10 times. The percentage means of bordering behaviour were compared using Student's t-test.

[1]The alpha value, corrected for multiple comparisons via the Bonferroni method, corresponds to 0.05/15 = 0.003.

Arrow in Fig.3B	Allele	Position Hybrid1 Assembly	Reference	Variant	Hybrid1 Gene ID	*C. elegans* 1:2:1 ortholog	Mutation Type	nt substitution
1	*tu667*	Contig6:2446422	A	T	Contig6-snap.265	*ifta-1*	nonsynonym [H>L]	CAT>CTT
2	*tu667*	Contig6:2446427	A	T	Contig6-snap.265	*ifta-1*	nonsynonym [N>Y]	AAC>TAC
3	*tu666*	Contig6:2448984	G	A	Contig6-snap.265	*ifta-1*	intronic_near_splice_site	-
4	*tu670*	Contig6:2449519	G	A	Contig6-snap.265	*ifta-1*	nonsense	TGG>TGA
5	*tu669*	Contig6:2454188	G	A	Contig6-snap.265	*ifta-1*	nonsense	TGG>TGA
6	*tu664*	Contig6:2460202	G	A	Contig6-snap.265	*ifta-1*	intronic_near_splice_site	-
7	*tu665*	Contig31:108694	C	T	Contig31-snap.23	*che-11*	nonsense	TGG>TGA
8	*tu675*	Contig31:107907	C	T	Contig31-snap.23	*che-11*	intronic_near_splice_site	-
9	*tu668*	Contig31:107639	G	A	Contig31-snap.23	*che-11*	nonsense	CAA>TAA
10	*tu663*	Contig10:2284105	T	A	Contig10-snap.384	*dyf-1*	nonsense	TAT>TAA
11	*tu678*	Contig56:591220	C	T	Contig56-snap.118	*xbx-1*	intronic_near_splice_site	-
12	*tu676*	Contig56:591849	C	T	Contig56-snap.118	*xbx-1*	nonsense	TGG>TAG

Supplementary Table S5. RS2333 social mutants isolated in forward genetics screens by means of EMS mutagenesis. Numbers in the first column correspond to the position of the mutation in the genes (see Fig. 2b).

Allele	Gene	Mutation	Type of mutation	Strain
tu1045	*ifta-1*	7 bp deletion	nonsense	RS2333
tu1044	*ifta-1*	5 bp insertion	nonsense	RS2333
tu1123	*ifta-1*	15 bp deletion + 7 bp insertion	nonsense	RS2333
tu1085	*che-11*	10 bp deletion + 11 bp insertion	nonsense	RS2333
tu1086	*che-11*	10 bp deletion + 2 bp insertion	nonsense	RS2333
tu1087	*che-11*	5 bp insertion	nonsense	RS2333
tu1081	*xbx-1*	6 bp deletion + 3 bp insertion	in frame	RS2333
tu1072	*dyf-1*	5 bp deletion	nonsense	RS2333
tu1074	*dyf-1*	25 bp deletion + 2 bp insertion	nonsense	RS2333

Supplementary Table S6. Mutant alleles of *ifta-1*, *che-11*, *dyf-1* and *xbx-1* orthologs in *P. pacificus* produced using CRISPR/Cas9 system. For each allele, the length of the genetic lesion, the type of mutation and the background strain from which the mutant was produced are indicated.

x	y	mean x	mean y	mean difference	t	df	*P*-value[1]	significance
RS2333	*tu1045*	34.83	96.11	61.28	-5.68	11	< 0.001	**
RS2333	*tu1044*	34.83	91.67	56.84	-5.25	11	< 0.001	**
RS2333	*tu1123*	34.83	96.67	61.84	-5.27	11	< 0.001	**
RS2333	*tu1085*	34.83	96.11	61.28	-5.67	11	< 0.001	**
RS2333	*tu1086*	34.83	96.11	61.28	-5.69	11	< 0.001	**
RS2333	*tu1087*	34.83	93.33	58.50	-5.41	11	< 0.001	**
RS2333	*tu1081*	34.83	96.11	61.28	-5.67	11	< 0.001	**
RS2333	*tu1072*	34.83	96.11	61.28	-5.68	11	< 0.001	**
RS2333	*tu1074*	34.83	93.33	58.50	-4.41	11	< 0.001	**

Supplementary Table S7. Statistical values from pairwise comparisons for mean bordering between wild-type strains and the mutant alleles of *ifta-1*, *che-11*, *dyf-1* and *xbx-1* homologues in *P. pacificus* generated by CRISPR/Cas9 system. Each strain/mutant was measured 10 times. The percentage means of bordering behaviour were compared using Student's t-test.

[1]The alpha value, corrected for multiple comparisons via the Bonferroni method, corresponds to 0.05/9 = 0.0055.

Allele	Gene	Mutation	Type of mutation	Strain
tu1097	*che-3*	4 Deletions + 14 Insertions	nonsense	RS2333
tu1096	*che-3*	7 Deletions	nonsense	RS2333
tu1100	*che-3*	15 Deletions + 8 Insertions	nonsense	RS2333
tu1098	*dyf-2*	2 Deletions	nonsense	RS2333
tu1102	*dyf-2*	9 Deletions + 19 Insertions	nonsense	RS2333
tu1099	*osm-12*	6 Deletions + 11 Insertions	nonsense	RS2333
tu1129	*osm-1*	6 Deletions + 4 Insertions	nonsense	RS2333
tu1090	*osm-3*	4 Deletions	nonsense	RS2333
tu1091	*osm-3*	4 Insertions	nonsense	RS2333
tu1092	*osm-3*	5 Deletions	nonsense	RS2333
tu1131	*klp-20*	8 Deletions	nonsense	RS2333

Supplementary Table S8. Mutant alleles of *che-3*, *dyf-2*, *osm-1*, *osm-3*, *osm-12* and *klp-20* orthologs in *P. pacificus* produced using CRISPR/Cas9 system. For each allele, the length of the genetic lesion, the type of mutation and the background strain from which the mutant was produced are indicated.

x	y	mean x	mean y	mean difference	t	df	*P*-value[1]	significance
RS2333	*tu1097*	34.83	95.56	60.73	-5.63	11	< 0.001	**
RS2333	*tu1096*	34.83	96.67	61.84	-5.75	11	< 0.001	**
RS2333	*tu1100*	34.83	95.56	60.73	-5.62	11	< 0.001	**
RS2333	*tu1098*	34.83	93.33	58.50	-5.40	11	< 0.001	**
RS2333	*tu1102*	34.83	95.56	60.73	-5.62	11	< 0.001	**
RS2333	*tu1099*	34.83	90.56	5573	-5.14	11	< 0.001	**
RS2333	*tu1129*	34.83	93.33	58.50	-5.42	11	< 0.001	**
RS2333	*tu1090*	34.83	34.44	0.39	0.03	11	0.975	ns
RS2333	*tu1091*	34.83	25.56	9.27	0.83	11	0.424	ns
RS2333	*tu1092*	34.83	28.89	5.94	0.52	11	0.612	ns
RS2333	*tu1131*	34.83	33.33	1.50	0.14	11	0.895	ns
RS2333	*tu1190;tu1131*	34.83	98.33	63.5	-5.90	11	< 0.001	**

Supplementary Table S9. Statistical values from pairwise comparisons for mean bordering between wild-type strains and the mutant alleles of the *che-3*, *dyf-2*, *osm-1*, *osm-3*, *osm-12*, *klp-20* homologues in *P. pacificus* generated by CRISPR/Cas9 system plus the *osm-3;klp-20* double mutant. Each strain/mutant was measured 10 times. The percentage means of bordering behaviour were compared using Student's t-test.

[1]The alpha value, corrected for multiple comparisons via the Bonferroni method, corresponds to 0.05/12 = 0.002.

x	y	mean x	mean y	mean difference	t	df	*P*-value[1]	significance
RS2333	*tu1100*	-0.984	-0.217	0.767	-8.339	4	0.0011	*
RS2333	*tu1085*	-0.984	-0.033	0.951	-128.14	4	< 0.001	***
RS2333	*tu1072*	-0.984	0.062	1.046	-15.231	4	< 0.001	**
RS2333	*tu1098*	-0.984	-0.254	0.730	-23.82	4	< 0.001	***
RS2333	*tu1045*	-0.984	-0.097	0.887	-13.036	4	< 0.001	**
RS2333	*tu1131*	-0.984	-0.970	0.014	-0.624	4	0.5662	ns
RS2333	*tu1129*	-0.984	-0.512	0.472	-4.140	4	0.0144	ns
RS2333	*tu1090*	-0.984	-0.080	0.904	-6.923	4	0.0023	*
RS2333	*tu1099*	-0.984	-0.261	0.723	-11.143	4	< 0.001	**
RS2333	*tu1081*	-0.984	-0.644	0.340	-6.196	4	0.0035	*
RS2333	*tu1190;tu1131*	-0.984	-0.036	0.948	-21.988	4	< 0.001	***

Supplementary Table S10. Student's t-test results from pairwise comparisons for 1-Octanol avoidance of RS2333 and the IFT-related mutants alleles generated in this study calculated from chemotaxis indexes (see methods).

[1] The alpha value, corrected for multiple comparisons via the Bonferroni method, corresponds to 0.05/11 = 0.0045

a							
**strain/mutant**	**mean 21% [O_2]**	**mean 10% [O_2]**	**mean difference**	**t**	**df**	***P*-value[1]**	**significance**
RS2333	31.11	11.67	19.44	2.74	4	0.052	ns
ifta-1(*tu1045*)	97.22	38.33	58.89	9.52	4	< 0.001	**
che-11(*tu1085*)	94.36	36.67	57.69	13.32	4	< 0.001	**
xbx-1(*tu1081*)	94.44	37.78	56.66	27.26	4	< 0.001	***
dyf-1(*tu1072*)	96.11	34.44	61.67	15.25	4	< 0.001	**
osm-3;klp-20	85.00	48.89	36.11	11.67	4	< 0.001	**
RSB001	87.78	17.22	70.56	7-10	4	0.002	*
**b**							
**strain/mutant**	**mean 10% [O_2]**	**mean 21% [O_2]**	**mean difference**	**t**	**df**	***P*-value[1]**	**significance**
RS2333	11.67	22.22	10.55	-1.14	4	0.317	ns
ifta-1(*tu1045*)	38.33	91.11	52.78	-7.66	4	0.002	*
che-11(*tu1085*)	36.67	96.11	59.44	-14.84	4	< 0.001	**
xbx-1(*tu1081*)	37.78	97.22	59.44	-37.83	4	< 0.001	***
dyf-1(*tu1072*)	34.44	93.33	58.89	-14.29	4	< 0.001	**
osm-3;klp-20	48.89	95.56	46.67	-16.47	4	< 0.001	***
RSB001	17.22	91.11	73.89	-7.44	4	0.002	*

Supplementary Table S11. Statistical values from pairwise comparisons for mean bordering at different oxygen concentrations ([O_2]) for RS2333, RSB001 and the mutant alleles of *ifta-1*, *che-11*, *dyf-1* and *xbx-1* homologues in *P. pacificus* produced using CRISPR/Cas9 system. (**a**) Oxygen shift from 21% to 10%. (**b**) Oxygen shift from 10% to 21%. The percentage means of bordering at each oxygen concentration were compared using Student's t-test.

[1]The alpha value, corrected for multiple comparisons via the Bonferroni method, corresponds to 0.05/14 = 0.0036.

**Strain/Mutant**	**mean 20% [O_2]**	**mean 21% [O_2]**	**mean difference**	**t**	**df**	***P*-value[1]**	**significance**
RS2333	0.007	0.014	0.007	-3.273	10	0.008	*
che-3(*tu1100*)	0.008	0.018	0.010	-6.248	10	< 0.001	**
che-11(*tu1085*)	0.010	0.016	0.006	-3.288	10	0.008	*
dyf-1(*tu1072*)	0.006	0.012	0.006	-9.495	10	< 0.001	***
dyf-2(*tu1098)*	0.010	0.018	0.008	-3.561	10	0.005	ns
ifta-1(*tu1045*)	0.007	0.017	0.010	-6.269	10	< 0.001	**
klp-20(*tu1131*)	0.006	0.009	0.003	-3.427	10	0.006	ns
osm-1(*tu1129*)	0.008	0.019	0.011	-8.564	10	< 0.001	***
osm-3(*tu1090*)	0.008	0.016	0.008	-4.772	10	< 0.001	**
osm-12(*tu1099*)	0.005	0.019	0.014	-6.116	10	< 0.001	**
xbx-1(*tu1081*)	0.008	0.018	0.010	-5.290	10	< 0.001	**
osm-3(*tu1190*)*;klp-20*(*tu1131*)	0.006	0.010	0.004	-3.574	10	0.005	ns

Supplementary Table S12. Ω-turn rates in response to oxygen concentration ([O_2]) shifts from 20% to 21% on food of RS2333 and the IFT-related mutants alleles generated in this study. The statistical values from pairwise comparisons between mean Ω-turns before and after oxygen shifts were calculated with the Student's t-test.

[1] The alpha value, corrected for multiple comparisons via the Bonferroni method, corresponds to 0.05/12 = 0.0042.

Designation	Gene	Strand	Sequence (5' to 3')
15456	*che-11*	sense	GAG AAC ACT TCA GGT GAG AGA C
15457	*che-11*	antisense	AAA ATA TTA ATG AGT ACC TCT TGC TG
15539	*che-3*	sense	AGT TTA TCA GAT GCC ATC TTT AGC
15540	*che-3*	antisense	GCC GCT GAG TAG ATC TGA TTG
15446	*dyf-1*	sense	CAA GTT TCC TTA GCA ACG G
15447	*dyf-1*	antisense	ACC GAT GGA GCT TTC TGT A
15528	*dyf-2*	sense	CAG GTG TAT CAG TTC CAA TGC
15529	*dyf-2*	antisense	CCA GCA GGT TTT TGT GAC A
Jim265.1F3	*ifta-1* exon 2	sense	GTT GGC AAT GCA AAC AAT TGC
Jim265.1R5	*ifta-1* exon 2	antisense	CTA GTG ATC TAG CTA TGC ATA TG
Jim265.2F3	*ifta-1* exon 15	sense	CCG GTT CCG GCT TCC GGA CTT G
Jim265.2R5	*ifta-1* exon 15	antisense	GCC AAA CGC CGT CCC AAT CAC
15559	*klp-20*	sense	GAA GTG AGG GAT CTA TTG GC
15560	*klp-20*	antisense	TCA GAT TTT ATG ATT GTG AAT ACT CA
sier0189	*npr-1* exon 1	sense	TCC GTA TGC AGT CTA TAT GTA CGT GG
sier0190	*npr-1* exon 1	antisense	CAC TCT CCA GTG CAT GGG AAC
sier0239	*npr-1* exon 6	sense	ACT AGA AGT CAT ACA TCC TTC C
sier0238	*npr-1* exon 6	antisense	TGT GCT TCA AAG TCA CGT
15549	*osm-1*	sense	GTG TAA TAA CTG AGT TGA TGT CAT TG
15552	*osm-1*	antisense	CAA TGC CTA GCC CAA ATA G
15533	*osm-12*	sense	GAG GCA TCG ACT GTT ACA CAT
15536	*osm-12*	antisense	CCT TCC CGA GAA TTA CAT CC
15520	*osm-3*	sense	TGC ACG TGT GTC ATA ATG TTA
15519	*osm-3*	antisense	AGT TTT CGA TTG CCG TTC
15458	*xbx-1*	sense	TGG AAT ATC TCT ATG CGA GG
15459	*xbx-1*	antisense	TAT TGA CTG CCA CTC ACC T

Supplementary Table S13. Primers for detecting gene lesions, produced by CRISPR/Cas9 system, in this study.
